# Circulatory Arrest, Brain Arrest and Death Determination

**DOI:** 10.3389/fcvm.2018.00015

**Published:** 2018-03-13

**Authors:** Sam David Shemie, Dale Gardiner

**Affiliations:** ^1^Division of Critical Care, Montreal Children’s Hospital, Research Institute of the McGill University Health Centre, Montreal, QC, Canada; ^2^Deceased Organ Donation, Canadian Blood Services, Ottawa, Ontario, Canada; ^3^Adult Intensive Care Units, Nottingham University Hospitals NHS Trust, Nottingham, United Kingdom; ^4^NHS Blood and Transplant, Bristol, United Kingdom

**Keywords:** death, brain death, cardiac arrest, circulatory arrest, organ donation, DCD, transplantation

## Abstract

Technological advances, particularly in the capacity to support, replace or transplant failing organs, continue to challenge and refine our understanding of human death. Given the ability to reanimate organs before and after death, both inside and outside of the body, through reinstitution of oxygenated circulation, concepts related to death of organs (e.g. cardiac death) are no longer valid. This paper advances the rationale for a single conceptual determination of death related to permanent brain arrest, resulting from primary brain injury or secondary to circulatory arrest. The clinical characteristics of brain arrest are the permanent loss of capacity for consciousness and loss of all brainstem functions. In the setting of circulatory arrest, death occurs after the arrest of circulation to the brain rather than death of the heart. Correspondingly, any intervention that resumes oxygenated circulation to the brain after circulatory arrest would invalidate the determination of death.

## Introduction

Technological advances, particularly in the capacity to support, replace or transplant failing organs, continue to challenge and refine our understanding of human death. Discussions about death are complex and deeply sensitive. All human beings die, yet there are philosophical, religious and cultural differences in the concept and definitions of death; and the loss of a loved one has profound emotional, psychological and spiritual impact on family and friends. These opinions are sometimes aired in academia and the media, where the discourse suffers from well-known deficits in understanding and/or awareness of the issues surrounding death determination.

A key modern challenge, that is a direct result of technological advances in the fields of resuscitation and transplant medicine, is the unavoidable relationship between death and deceased organ donation. Death determination requires timely and exacting criteria. Despite the diversity of perspective, it is important to reconcile how dying is, first and foremost, a biological process and the declaration of death an event in this biological process. This paper will advance the rationale for the continuing evolution towards a single unified determination of death, an event which can be diagnosed on permanent brain arrest, that occurs subsequent to a devastating brain injury or circulatory arrest.

## The Biology of Dying and Death

Over the last 60 years, advances in medicine and technology have enriched our understanding of the biology of life and death. The medical specialties that have improved our understanding include: cardiopulmonary resuscitation (CPR) and physiology; mechanical ventilation; cardiac surgery and cardiopulmonary bypass; intensive care unit (ICU) based life support; extracorporeal support and extracorporeal membrane oxygenation (ECMO); cell biology; and organ donation, preservation and transplantation. These developments have globally saved countless lives. They have also informed how medicine and society understands what it means to be alive, dying or dead.

**Figure 1:**
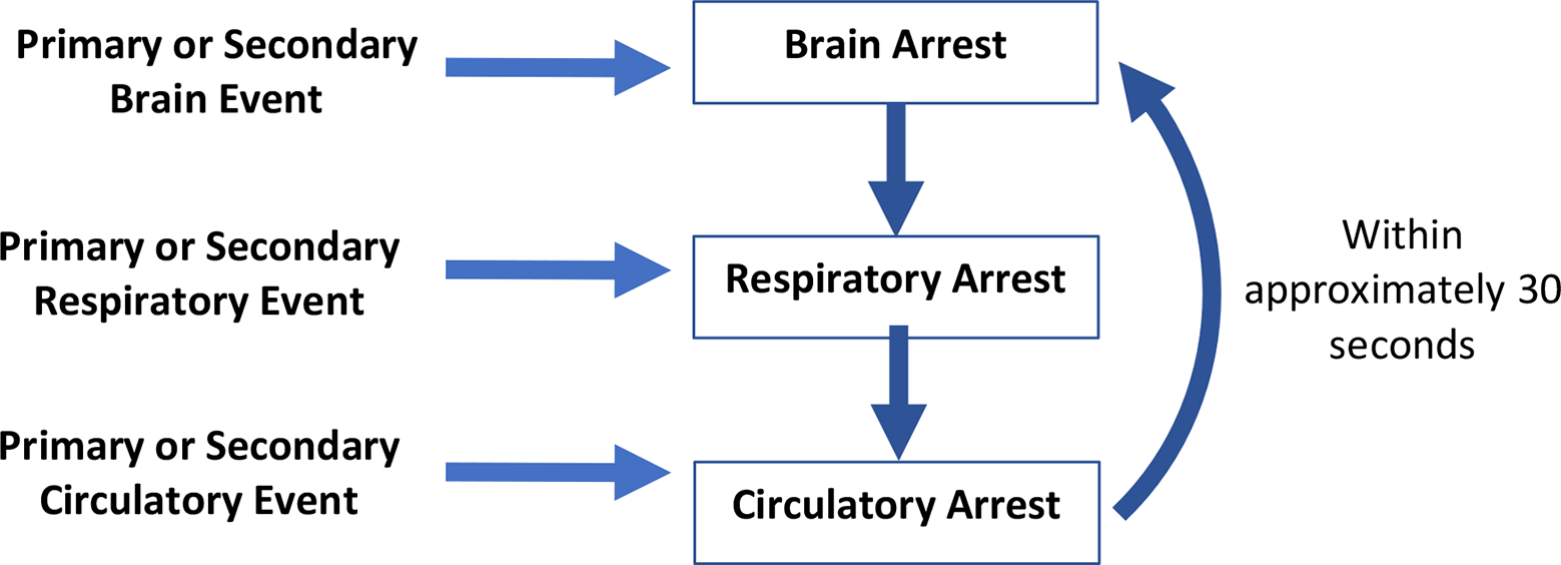
Mechanisms which might precipitate organ arrest and the dying process.

The dying process, which can be interrupted by various forms of intra and extracorporeal life-sustaining treatment, is sequential and predictable. In general, death occurs by one of three physiological mechanisms (1) (Figure 1):

1. Primary or secondary brain event with cessation of brain function, most often associated with intracranial hypertension and cessation of brain blood flow, leading to apnea, hypoxemia, cardiac arrest and cessation of circulation.
2. Primary or secondary respiratory event causing hypoxemia resulting in cardiac arrest and cessation of circulation to all organs including the brain.
3. Primary or secondary cardiac event resulting in cardiac arrest and cessation of circulation to all organs including the brain.

Sustaining life is based on delivering oxygen and nutrients to the mitochondria of cells, providing energy required for metabolic processes and cell life. Each organ is comprised of billions to trillions of cells and the interdependent multi-organ function is sustained by interconnectivity principally through the vascular system. Organ function can be sustained or reanimated in the following technology dependent scenarios that are all fundamentally predicated on oxygenated circulation:

1. Living patients with oxygenated circulation supported by:
  - Intrinsic cardiopulmonary support (e.g., mechanical ventilation, hemodynamic support of cardiovascular function).
  - Extrinsic extracorporeal support [e.g., ventricular assist devices, extracorporeal membrane oxygenation (ECMO)].
2. Dead persons with oxygenated circulation supported by:
  - Intrinsic cardiopulmonary support (e.g., somatic support after neurologically determined death during organ donor management or pregnancy).
  - Extrinsic extracorporeal support [e.g., normothermic regional perfusion in controlled or uncontrolled donation after circulatory death (DCD), ECMO following neurologically determined death].
3. Individual organs supported outside the body with oxygenated circulation (e.g., *ex-situ*/*ex-vivo* organ support).

It is an immense achievement that modern medicine can support oxygenated circulation in situations that include complete absence of intrinsic cardiopulmonary function. One can reasonably foresee the ability to provide oxygenated circulation to living patients with extracorporeal support while sustaining and repairing their individual organs with *ex-situ* support followed by auto-transplantation.

Scientific endeavor continues to push at the margins of human viability. It has also accelerated the change in the prevailing understanding of the essential aspect of human death. A journey of conceptual and scientific discovery that has led us from breathing to circulation and onto the brain. Brain function—not heart or circulatory function—has emerged as the critical margin between life and death.

## Dead Donor Rule

The *dead donor rule* has been the central ethical, moral and legal requirement that has guided transplantation (2). The balance between minimizing donor organ damage while adhering to the dead donor rule has driven the need for timely and exacting criteria for death determination. Calls to abandon the dead-donor rule (3) highlight the ethical, philosophical and scientific complexities of defining the moment of death. They are often linked to claims that donation of vital single organs should be able to occur during the dying process prior to death, as this would lead to more organs available for transplant. It can be acknowledged that ongoing complexities in death determination fuel a theoretical merit for proposals to redefine organ donation eligibility to a point before death. However, they ignore the inaccuracies of prognostication in predicting those who inevitably will die, which pose troubling clinical challenge and risk of abuse. In a voluntary system of organ donation based on public and professional trust, the dead donor rule remains an indispensable ethical protection for dying patients (4).

## Death and the Cessation of Brain Function

Three major advances in health care in the 1960’s have led to the concept that the cessation of brain function constitutes death. Firstly, the development of intensive care units with artificial airways and mechanical ventilators allowed patients with irreversible apnea to be maintained, thus interrupting the natural progression from brain arrest to cardiac arrest. Secondly, the more routine availability of cardio-pulmonary bypass for cardiac surgery allowed the circulation of patients with cardioplegic arrest to be maintained, interrupting the natural progression from cardiac arrest to brain arrest. Thirdly, the arising new discipline of transplant surgery required ethical and legal clarity over the determination of death.

Prior to the evolution of resuscitative measures and the introduction of mechanical ventilators in the mid-20th century, death was determined either by external signs of death (e.g., rigor mortis, putrefaction) or predominantly cardio-respiratory signs of death. The attachment to the heartbeat as being central to life was founded on the discovery of blood circulation by William Harvey in 1628 and the stethoscope by Laennec in 1816. Bouchut in the 19th Century (5), gave greater confidence to the confirmation of absent circulation (notoriously difficult when the pulse is slow and weak) by advocating the routine use of the stethoscope.

It was however even earlier, when Maimonides (6), a 12th century rabbi and physician scholar, suggested that the brain through control of breathing was of primary importance in sustaining life. He concluded that decapitated individuals were deceased at the time of decapitation not when their circulation ceased. For 800 years Maimonides conclusion lay dormant until the clinical appearance of brain death was first described in a seminal work by the French physicians Mollaret and Goulon in 1959. They described the mechanically ventilated patients they were observing as *coma dépassé* meaning *“a state beyond coma*” (7). The 1968 Ad Hoc Committee of the Harvard Medical School undertook to define irreversible coma as a new criterion for death (8). They established a new, neurologically-based definition of death, defined as “*unresponsiveness and lack of receptivity, the absence of movement and breathing, the absence of brain-stem reflexes and coma whose cause had been identified”*. The original text remains relevant and includes “from ancient times, when the respiration and heart stopped, the brain would die in a few minutes”; “in those times, the heart was considered to be central organ of the body”; “this is no longer valid when modern resuscitative and supportive measures are used”; “characteristics of a *permanently* nonfunctioning brain; no discernable central nervous system activity”. In turn the US Uniform Determination of Death Act (9) codified the whole-brain formulation in stating that “*an individual who has sustained irreversible cessation of all functions of the entire brain, including the brainstem, is dead*”. This formulation is the one most commonly applied worldwide and forms the foundation for legal codification in many Western nations.

Brain death is the final endpoint of extreme brain injury and is better characterized as *brain arrest* (10) which is the complete and permanent cessation of consciousness, capacity to breathe and brainstem reflexes. The most common mechanism for primary brain arrest is refractory intracranial hypertension that exceeds arterial inflow pressure leading to cessation of brain blood flow. As long as mechanical ventilatory support is maintained, the heart will continue to function, thus sustaining oxygenated circulation to the body even when there is complete loss of circulation to the brain.

## Death and Cessation of Circulation

While doctors have been declaring death for centuries criteria for death determination after cardiac arrest were rarely formalized, and ranged from absence of movement, breathing, heart sounds, pulse or ECG activity. Current practices remain highly variable and inconsistent (11).

The ability to restore circulation with cardiopulmonary resuscitation, and even more so with extracorporeal support or reanimate heart function with *ex-situ* support, defies historical concepts of death determination based on the simple detection of heart and circulatory cessation. Effectively, modern in-hospital death after cardiac arrest is contingent on the consideration of CPR and ECMO to circulate oxygen – indications for use, availability and decisions at the end-of-life. In advanced health care systems, death can only occur after CPR and/or ECMO is either discontinued or not provided.

These realities are germane to criticisms of death determination in DCD. Here the complexities of science and philosophy entangle within debates around the minimal period of observation before death can be declared (see the below section on auto-resuscitation), whether loss of circulatory function is not “irreversible” within the time limits proposed and whether death is related to arrest of heart function or arrest of circulation. Given the ability to reanimate the heart by *ex-situ* oxygenated circulation this infers that the heart is not “dead”, but it is unable to sustain circulation in the body of origin. It is therefore not the arrest of heart function, but the absence of circulation (intrinsic or extrinsic) that determines death after cardiac arrest. The debate between defining the term irreversible as meaning “*cannot be reversed under any circumstances*” versus permanent where circulation “*will not reverse under existing circumstances*” (12, 13) is generally confined to academia. Most clinicians responsible for the declaration of death appear to accept in practice the permanence standard (14). The Institute of Medicine (15) and the ethics committee of the American College of Critical Care Medicine (16) take a similar pragmatic view to the ambiguity surrounding the term “irreversible”. Fundamentally, and most relevant to DCD, is not whether the body or brain circulation and function can be resumed (because it can), but rather, whether it will be. This is contingent on the preceding CPR and ECMO decisions.

## Brain Blood Flow and Brain Arrest

**Figure 2:**
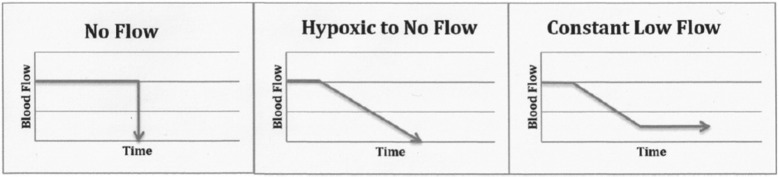
Mechanism of brain ischemia. For illustrative purpose only, in order to demonstrate the various clinical contexts that were identified in the articles identified in this review. It is understood that values for blood flow and times, as well as the slope will vary according to each clinical situation (17).

Circulatory arrest leads to absent brain blood flow which in turn leads rapidly to loss of brain function. Figure 2 illustrates models of circulatory arrest (abrupt no-flow such as ventricular fibrillation; hypoxic to no-flow such as withdrawal of mechanical ventilation in controlled DCD; constant low-flow such as CPR or shock states) (17).

Consciousness and EEG activity is lost within 30 s after abrupt circulatory arrest (17)(Figure 1). Cessation of brain function with isoelectric EEG may occur *prior* to circulatory arrest in hypoxia states (18), such as after withdrawal of mechanical ventilation in controlled DCD (19). If oxygenated circulation does not resume, then beyond 30 s the brain will remain in a state of permanent non-function. This evidence supports a key physiological justification for the legitimate adherence to the dead donor rule in controlled DCD.

If however brain blood flow is quickly re-established, spontaneously or with interventions such as CPR or ECMO, then brain function may resume. It is not clear how long brain blood flow must have ceased to uniformly preclude reanimation of brain function with restoration of circulation. While it is commonly stated that the normothermic brain cannot be revived to normal function after more than 8 to 10 min of complete ischemia (20), the duration of circulatory arrest required for brain function to cease irreversibly in response to a resumption of circulation in humans is modifiable by peri-arrest conditions and varies in animal models. The lower limit of cerebral blood flow required to sustain neuronal viability is 100 ml/min/100 g in animals (21) but in humans, the lower limit of brain blood flow that results in global loss of neuronal function requires further investigation. These findings have relevance to uncontrolled DCD, where cardiac compressions and mechanical ventilation (and thus oxygenated circulation) may be reinstituted after failed CPR and termination of resuscitation efforts. Reanimation of brain function and consciousness under these circumstances have been reported (22).

## Autoresuscitation

The clinical distinction between cardiac electrical activity and mechanical function of the heart is important in DCD. Physiologically, it is no surprise that ECG activity can persist for many minutes after terminal cardiac arrest without circulation (23, 24). It is heart’s ability to generate circulation, not electricity, that is of key importance to declaration of death.

There have been case reports of spontaneous, unassisted resumption of heart function after cardiac arrest, so called autoresuscitation (AR), ranging from seconds to minutes (25, 26). While AR is rare, the true incidence is unclear. Reports are hampered by variability in monitoring techniques and observation, and thus some cases may be related to diagnostic error. AR appears to be reported more frequently and for longer periods of time after failed CPR, as may apply to uncontrolled DCD, than after withdrawal of life support (no CPR provided) as applicable to controlled DCD. In a retrospective study of 73 controlled DCD patients no patients exhibited AR during the five-minute observation period following asystole (27). A prospective pilot study of ICU deaths after withdrawal of life support showed no evidence of AR beyond 89 s, well within the generally recommended 2–5 min observation period (24). A larger prospective study is underway to confirm these preliminary results (28).

## Unified Brain-Based Determination of Death

A unified brain-based definition of death is where death is determined by the diagnosis of brain arrest, characterized by permanent loss of capacity for consciousness and loss of all brainstem functions. Following cardio-respiratory arrest, confirming circulatory arrest is the most valid method to confirm absence of brain blood flow, and hence brain arrest. Whereas in cases where the brain injury itself has caused brain arrest, yet the circulation is maintained by intensive care interventions, a clinical diagnosis of the loss of these brain functions determines death.

**Figure 3:**
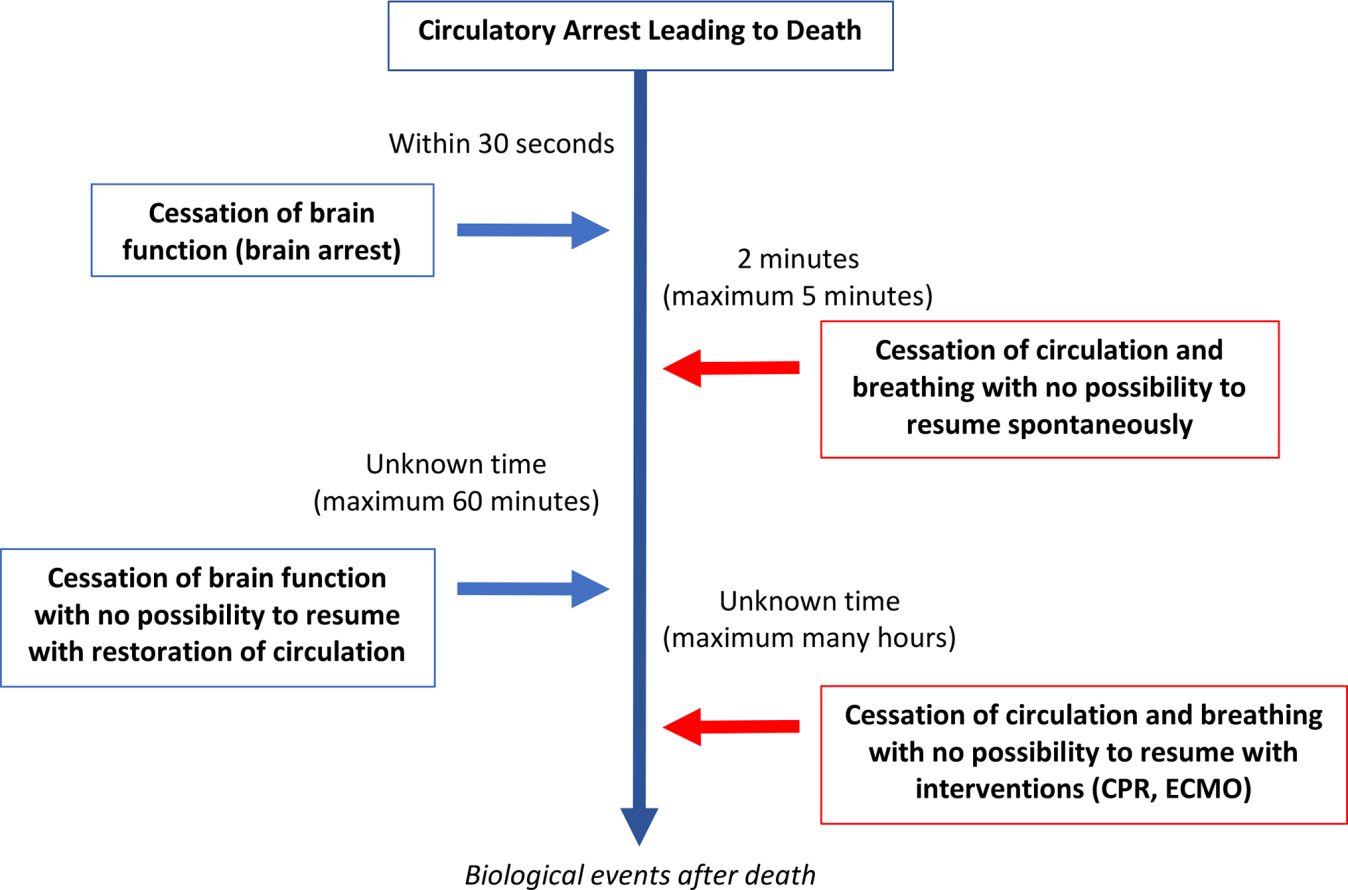
Physiological sequences in the dying process following circulatory arrest and where there has been a consensual decision to withdraw life-sustaining therapies (WLST) and to not provide cardiopulmonary resuscitation (CPR). Adapted from (29).

Figure 3 Illustrates the relationship between the cessation of brain function and circulatory arrest. (adapted from reference 29)

There are a number of interventions in DCD that are intended to minimize warm ischemic donor organ injury and improve transplantable graft function. These interventions can restore oxygenated circulation- in the body before death, in the body after death or in isolated *ex-vivo* organ systems. Post mortem cardiac compressions and mechanical ventilation (similar to CPR) and normothermic regional perfusion (similar to ECMO) provide oxygenated circulation to sustain donor organ function prior to transplant. Further investigation is required to define the lower limits of brain blood flow below which neuronal function cannot be maintained. In the absence of this clarity, if the predominance of brain function for determination of death (29, 30) achieves broader acceptance, then interventions that reinstitute *any* oxygenated circulation to the brain would invalidate the determination of death. While efforts may be made to surgically interrupt the arterial supply to the brain, the timing should occur prior to restarting the circulation and the effectiveness to preclude direct or collateral sources of brain blood flow should be verified.

## Conclusion

Given the ability to reanimate organs after death, both inside and outside of the body, through reinstitution of oxygenated circulation, concepts related to death of organs (e.g., cardiac or circulatory death) are no longer valid. This paper advances the rationale for a single conceptual determination of death related to permanent brain arrest, resulting from primary brain injury or secondary to circulatory arrest. In the setting of circulatory arrest, death occurs after the arrest of circulation to the brain rather than death of the heart. On this basis, the current practice of DCD is predicated.

## Author Contributions

Both authors drafted, reviewed and finalized the paper.

## Conflict of Interest Statement

All the authors declare that the research was conducted in the absence of any commercial or financial relationships that could be construed as a potential conflict of interest.
